# Gene Expression Variability in Human Hepatic Drug Metabolizing Enzymes and Transporters

**DOI:** 10.1371/journal.pone.0060368

**Published:** 2013-04-23

**Authors:** Lun Yang, Elvin T. Price, Ching-Wei Chang, Yan Li, Ying Huang, Li-Wu Guo, Yongli Guo, Jim Kaput, Leming Shi, Baitang Ning

**Affiliations:** 1 Division of Systems Biology, National Center for Toxicological Research, Food and Drug Administration, Jefferson, Arkansas, United States of America; 2 Department of Pharmaceutical Sciences, University of Arkansas for Medical Sciences, Little Rock, Arkansas, United States of America; 3 Department of Pharmaceutical Sciences, College of Pharmacy, Western University of Health Sciences, Pomona, California, United States of America; MOE Key Laboratory of Environment and Health, School of Public Health, Tongji Medical College, Huazhong University of Science and Technology, China

## Abstract

Interindividual variability in the expression of drug-metabolizing enzymes and transporters (DMETs) in human liver may contribute to interindividual differences in drug efficacy and adverse reactions. Published studies that analyzed variability in the expression of DMET genes were limited by sample sizes and the number of genes profiled. We systematically analyzed the expression of 374 DMETs from a microarray data set consisting of gene expression profiles derived from 427 human liver samples. The standard deviation of interindividual expression for DMET genes was much higher than that for non-DMET genes. The 20 DMET genes with the largest variability in the expression provided examples of the interindividual variation. Gene expression data were also analyzed using network analysis methods, which delineates the similarities of biological functionalities and regulation mechanisms for these highly variable DMET genes. Expression variability of human hepatic DMET genes may affect drug-gene interactions and disease susceptibility, with concomitant clinical implications.

## Introduction

Drugs are usually approved based on safety and efficacy data in a limited number of patients that are thought to represent the entire population. However, individuals in a population show differences in drug sensitivity, efficacy, toxicity, and dosing [1], [2], [3]. For the majority of drugs used in treating common diseases, only 25% to 60% of patients respond to a specific medication [2]. A widely cited article [4] stated that more than 2 million adverse drug reaction cases were reported annually in the United States, including approximately 100,000 instances of death.

The variability of drug responses among individuals in the population occurs because of complex, multifactorial contributions of genetic factors (such as single nucleotide polymorphisms and copy number variations), environmental factors (such as dietary components), disease/health condition of the individuals, and drug-drug interactions. These interactions alter drug absorption, metabolism and pharmacokinetics differently in patients, leading to interindividual variability of drug efficacy, safety, and adverse drug reactions [1], [2], [5]. Many studies have investigated associations among genetic polymorphisms of drug-metabolizing enzymes and transporters (DMETs) and drug responses: the number of drug-gene relationships deposited in PharmGKB (http://www.pharmgkb.org/) has grown to 24,329 up to date (September, 2011). Besides the polymorphisms in coding regions, gene expression variability is another contributor to interindividual differences in drug responses, which is difficult to study in humans. The US Food and Drug Administration (FDA) maintains a database (http://www.fda.gov/drugs/scienceresearch/researchareas/pharmacogenetics/ucm083378.htm) of genetic variants that affect the treatment outcomes of some drugs. However, these variations in coding sequences do not fully explain differences in drug responsiveness between individuals with similar variations. A better understanding of the interindividual variability in the expression of the DMETs is one of the fundamental requirements needed to improve drug efficacy and mitigate adverse reactions to empower personalized medicine.

Markedly high interindividual variability of DMET activities among humans was previously documented [6], [7]. Rodriguez-Antona et al. [8] observed large variations of 10 cytochrome (*CYP*) enzymes among 12 human liver samples with 40-fold differences in *CYP2C19*, 50-fold differences in *CYP3A4*, and more than 500-fold differences in *CYP2D6*. Analyses of gene expression of 261 DMETs in primary hepatocytes from 6 individuals showed that *GSTM5* had the greatest variation (166-fold), followed by *CYP26B1* (157-fold) and *SULT1C1* (58-fold) [9]. Reports of systematic analysis of the DMET expression spectrum in a larger sample size have been published. For example, a genome-wide expression quantitative trait loci (eQTL) study that aimed at mapping the genetic architecture of gene expression in human liver was performed in a cohort containing 427 human liver samples [10]. In addition, using the same set of human liver samples, Yang et al. systematically analyzed the full spectrum of functionality of *CYP* enzymes in human liver by profiling gene expression, protein activity, and genetic variants and their relationships. However, the expression patterns and interindividual variability of other DMETs in human liver were not analyzed in that study [11].

Other studies focusing on interindividual variability of DMET were limited by small sample sizes and/or a focus on a subset of hepatic DMETs [9], [11], [12], [13]. In order to evaluate the interindividual variability of hepatic DMET expression in a larger sampling of the human population, the current study archived the expression profiles from a published dataset containing of 427 human liver samples [10], and further analyzed interindividual variability focusing on of 374 DMET genes. Our results demonstrated a wide range of interindividual variability in the expression of human hepatic DMETs genes. Coexpression network analysis was also used to delineate biologically meaningful modules that suggest co-regulation among DMETs. Finally, the clinical implications of interindividual variability of DMETs in personalized medicine are discussed.

## Results

### Interindividual variability in expression for DMET genes is greater than non-DMET genes

**Figure 1:**
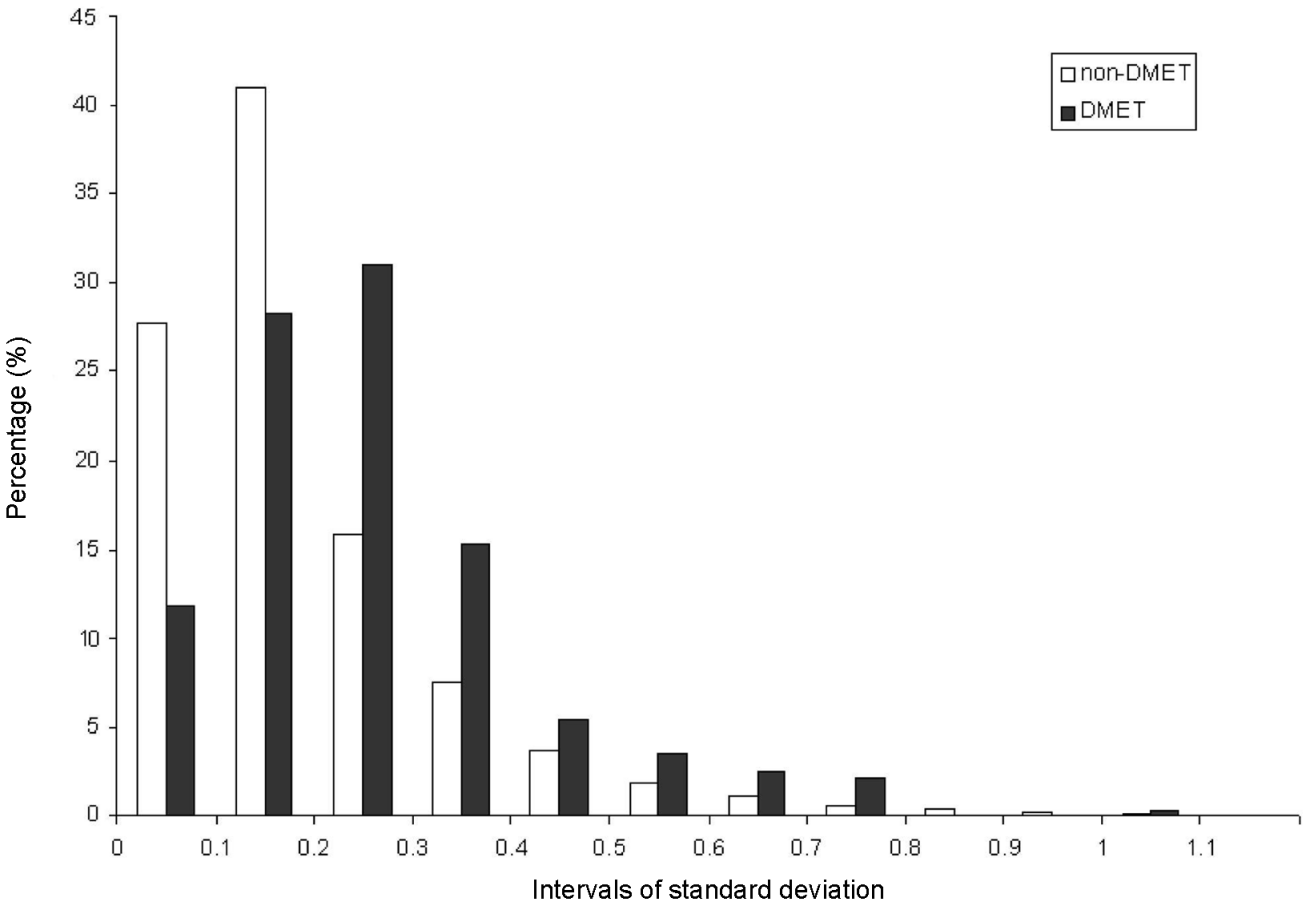
The standard deviation (SD) for the expression of DMET and non-DMET genes among 427 individuals. The DMET genes appear to have a higher likelihood of having high SD compared to non-DMET genes. The X-axis shows the SD interval and Y-axis represents the probability of 427 individuals with an indicated SD interval value.

The interindividual variability of 374 DMETs in a cohort of 427 human liver samples was evaluated by calculating the mean expression value, median expression value, standard deviation (SD), coefficient of variation (CV), the highest expression value(s) (Max) and the lowest value(s) (Min) (summarized in Table S1). Using SD as a measure of interindividual variability in expression, we compared 374 DMET genes against 19,167 genes in the data set that are not considered to be involved directly in drug metabolism and are designated as non-DMET genes for this study. The non-DMET genes (Figure 1) were more likely to distribute to the lower SD interval of 0 to 0.2, while the DMET genes were more likely to distribute to the higher SD interval of 0.25 to 1.1. The distribution of SD suggested that DMET genes have higher interindividual variability in expression than non-DMET genes. When the CV was used to evaluate the interindividual variability between DMETs and non-DMETs, a similar patter with SD distribution was observed (data unshown).

As a reference, 20 genes typically considered to be housekeeping genes were selected to compare the expression variability of DMET genes. These housekeeping genes are often used as controls due to their ubiquitous and stable expression across different biological conditions [14]. The majority of these 20 housekeeping genes had an SD value between 0.05 and 0.20, and none had an SD value higher than 0.25. This comparison indicated that DMET genes show much higher expression variability than housekeeping genes.

### Top 20 most variably expressed DMETs in human liver

**Figure 2:**
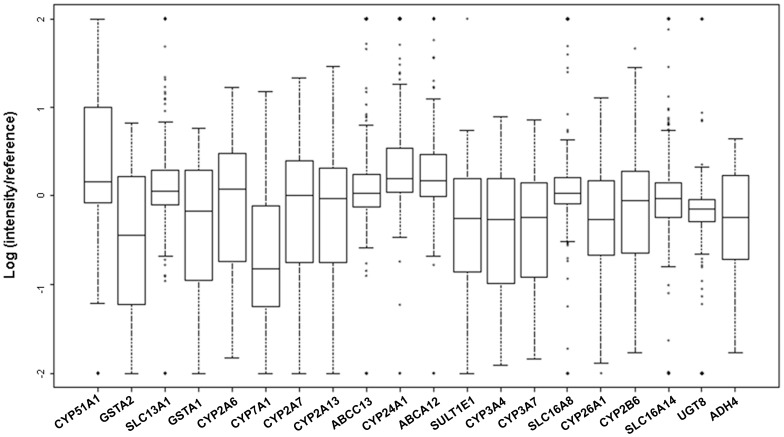
Box plot of the top 20 most variably expressed human hepatic DMET genes. The bottom and top of the boxes represent the 25th and 75th percentiles, respectively. The median is indicated by a bold line. The length of the box is the interquartile range (IQR). Values more than 1.5 IQRs are labeled as dots. The X-axis indicates names of DMETs, and the Y-axis indicates “Log10 Ratio of Intensity (samples/references). The reference is the pooled RNA derived from 192 liver samples selected fro sex balance from Vanderbilt and Pittsburgh samples [10].

The majority of the DMET genes exhibited large expression variability among the individuals in the study population, with some DMETs showing 1,000-fold difference between individuals. Shown in Figure 2 are 20 of the most variably expressed DMET genes in the liver as determined by the highest CV values. The median expression values are indicated as a bold line in the middle of each box, while the bottom and top of the box represent the 25th and 75th percentile, respectively. *CYP3A4*, *CYP2B6*, *CYP2A6*, *CYP3A7*, *GSTA1 and SULT1E1* are genes involved in the metabolism of many drugs and xenobiotics and had the highest variability in gene expression. In addition, transporter genes *SLC13A1, ABCC13*, *SLC16A8 and SLC16A14* were among these most variably expressed genes. Surprisingly, an important drug-metabolizing gene, *CYP2D6*, was not on the list of the top 20 most variably expressed genes in the current cohort of samples. Interindividual variability of human hepatic DMET expression levels within this population was demonstrated by the expression differences (fold changes) between the highest and the lowest expressing individuals. Table 1 lists the top 20 most variably expressed DMETs with their expression ranges (fold changes). “Expression Difference” indicates the fold differences between two individuals at the extremes of expression for that gene. The numbers in the column “Related Drugs” indicate the number of drugs metabolized by the corresponding genes (from the PharmGKB database www.pharmgkb.org). The analyses in this study were based on published Agilent two-color microarray data in which a cutoff standard between log_10_ values of −2 to 2 was used. Hence, some of the DMET genes with the highest variability, such as *ABCA12* and *UGT8*, showed fold changes over the detection standard. These are designated as O.E.R. (Over the Evaluation Range) in Table 1. Some DMET genes most commonly involved in drug metabolism which showed large and measurable interindividual variability in this population were:

**Table 1:** Interindividual Variability of the 20 Most Variably Expressed DMET Genes among 427 Subjects.

Gene Symbol	Highest Expression (log10 ratio)	Lowest Expression (log10 ratio)	Expression Difference (Fold Change)	Number of Related Drugs^a^
ABCA12	2	−2	O.E.R^b^	NA
ABCC13	2	−2	O.E.R	NA
ADH4	0.6378	−1.767	254	1
CYP24A1	2	−2	O.E.R	2
CYP26A1	1.108	−2	>1282	1
CYP2A13	0.8784	−1.384	183	6
CYP2A6	1.215	−1.819	1081	38
CYP2A7	1.258	−2	>1811	1
CYP2B6	1.565	−1.711	1888	57
CYP3A4	0.8979	−1.909	641	245
CYP3A7	0.8617	−1.842	505	22
CYP51A1	2	−2	O.E.R	2
CYP7A1	1.18	−2	>1513	5
GSTA1	0.7653	−2	>582	20
GSTA2	0.8187	−2	>659	6
SLC13A1	2	−2	O.E.R	NA
SLC16A14	2	−2	O.E.R	NA
SLC16A8	2	−2	O.E.R	NA
SULT1E1	2	−2	O.E.R	9
UGT8	2	−2	O.E.R	NA

a The number of the related drugs was derived from PharmGKB database.

b O.E.R stands for Over the Evaluation Range.

- *CYP3A4* (metabolizing 245 drugs) with a 641-fold difference between the highest and lowest expression levels.
- *CYP2B6* (metabolizing 57 drugs) with a 2,704-fold difference.
- *GSTA1* (metabolizing 20 drugs) with a 582-fold difference.

### Co-expression of human hepatic DMETs

**Figure 3:**
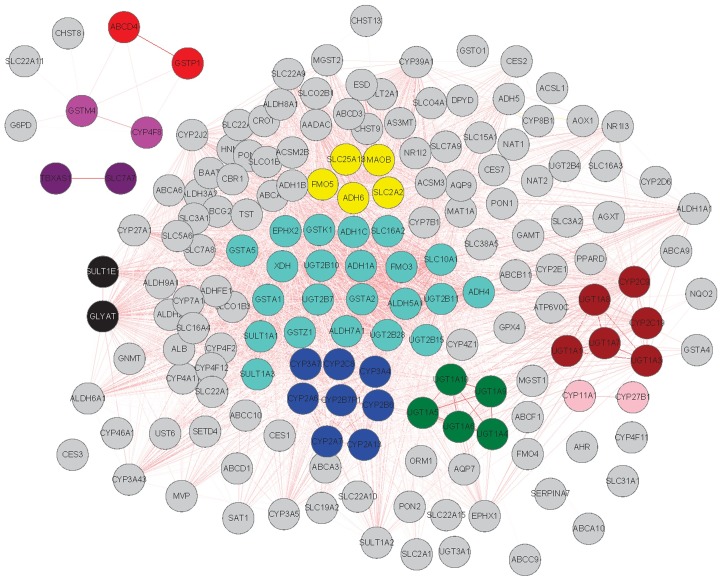
A topological overlap matrix (TOM) of all 374 DMET genes. Both the rows and the columns are sorted by hierarchical clustering. The colors specify the strength of the pair-wise topological connections (yellow: not significantly connected; red: highly connected). Genes that are highly connected within a cluster are defined as a module. Each module was assigned a unique color identifier (turquoise and blue), with the remaining genes colored gray.

**Figure 4:**
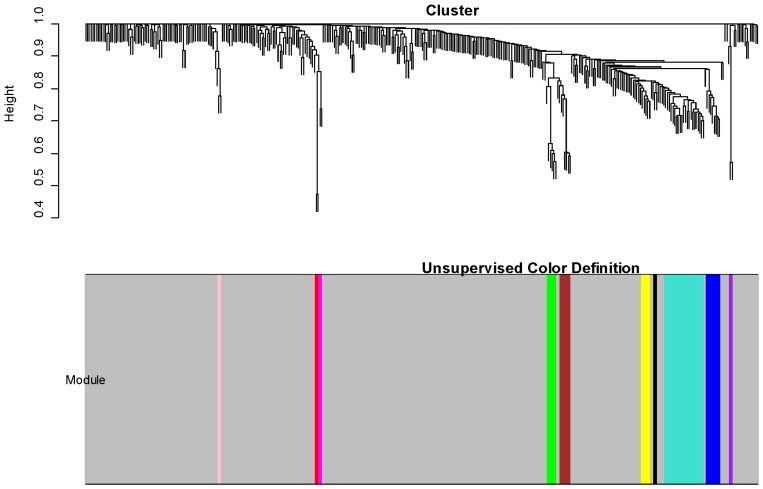
The visualization of the coexpression network for DMET genes. The graph highlights that genes in a liver coexpression network fall into 10 distinct modules, where genes within a module are more highly interconnected with each other than with genes outside the module.

Genes which are co-expressed potentially can be considered to be functionally related, since genes associated with specific biological processes usually are co-expressed [15], [16], [17] or involved in the same network. Gene co-expression network analysis aids in identifying genes interacting within and between modules [11], [15], [17]. Topological overlap matrix (TOM) analysis was performed to identify modules consisting of highly interconnected expression traits within the co-expression network for the 374 DMET genes. Ten distinct modules including 55 genes that were differentially expressed among individuals were identified (Figure 3). The remaining 319 DMET genes with differential expression levels failed to fall into any module. Genes within a module are usually co-expressed together with a higher correlation than genes outside of this module. The co-expression interactions between genes differentially regulated among individuals in one module and across modules are represented in a network (Figure 4). We interpret these modules in the co-expression networks as functional drug metabolic units. Detailed information for the contents of each module is listed in the Table S1. The largest module (turquoise color) contains 22 DMETs enriched with phase II enzymes including *UGT2B* family members, *GSTs* and *GSTs* and *SULTs*. Another module (blue color) contains *CYP3A4, CYP3A7, CYP2B6, CYP2C8* and additional *CYP* enzymes. These genes evolved to metabolize a broad range of xenobiotics and based on the analysis described here, possibly are co-regulated. Two other modules (green and brown) have many interactions at close distances indicating their high similarity in levels of co-expression. The most prominent members of these modules are *UGT1A* family members which may share common regulatory mechanisms. The *UGT1* complex locus posses a unique gene structure in which each family members have different promoters and N-terminal exons, but share identical exons in the C-terminal region of the gene [18]. This unique exon sharing process may partially explain the similar expression patterns of members for this gene family.

### Clinical implications of variably expressed DMETs in humans

Interindividual variability in the expression of hepatic DMETs among patients is one of the most important factors accounting for differences in drug metabolism, disease severity, and clinical effectiveness of drugs. DMET enzymes have been comprehensively characterized for their roles in metabolizing commercially available drugs. Using information derived from the PharmGKB database (http://www.pharmgkb.org/), the top 10 most clinically important DMETs were ranked according to the number of drugs they metabolize/eliminate. GeneGo database (www.genego.com) was searched with this gene list for related clinical therapeutics, biological pathways and associated diseases. As summarized in Table 2, the 10 most important genes showed highly variable expression and had significant clinical relevance to the metabolism/transport of endogenous molecules and/or xenobiotics. Moreover, potential impact of these genes on diseases, such as metabolic disorders and cancer development, were also revealed by GeneGo analysis.

**Table 2:** Expression Variability of Top 10 Most Important DMETs and Their Biological Significances.

Gene Symbol	Maximum Expression (log10 intensity)	Minimum Expression (log10 intensity)	Expression Difference (Fold change)	Classification based on major substrate (Xenobiotics or nonxenobiotics)	Related Drugs	Associated Diseases
CYP 2B6	1.565	−1.711	1888	Xenobiotics	**31 drugs:** Amiodarone, Sulconazole, Clopidogrel, Toremifene, Efavirenz, Phenytoin, Bupropion, Retinoic acid, Orphenadrine, Benidipine, etc.	**3 diseases:** Stomach Neoplasms, Breast Neoplasms, Prostatic Neoplasms
CYP2C19	1.738	−1.165	800	Xenobiotics	**67 drugs:** Ketoconazole, Amiodarone, Rabeprazole, Sulconazole, Tegaserod, Cimetidine, Clopidogrel, Latrepirdine, RO3201195, Proguanil, etc.	**1 disease:** Breast Neoplasms
CYP3A4	0.8979	−1.909	641	Xenobiotics	**138 drugs:** Ketoconazole, Amiodarone, Octreotide, Indinavir, Metronidazole, Domperidone, Manidipine, Rabeprazole, Glibenclamide, Oxcarbazepine, etc.	**6 diseases:** Carcinoma, Ductal, Breast Colorectal Neoplasms, Drug Toxicity, Breast Neoplasms, Prostatic Neoplasms
CYP2C8	0.8525	−1.566	262	Xenobiotics	**60 drugs:** Ketoconazole, Amiodarone, Indinavir, Lovastatin, Trimethoprim, Desipramine, Tegaserod, Clopidogrel, Candesartan cilexetil, etc.	**1 disease:** Breast Neoplasms
CYP3A5	0.7256	−1.324	112	Xenobiotics	**15 drugs:** Ketoconazole, Amprenavir, Troleandomycin, Phenytoin, Clarithromycin, Nelfinavir, Rifampicin, Erythromycin, Diltiazem, Tanespimycin, etc.	**6 diseases:** Stomach Neoplasms, Carcinoma, Ductal, Breast Colorectal Neoplasms, Breast Neoplasms, Prostatic Neoplasms
UGT1A1 (UD11)	0.7943	−1.177	94	Xenobiotics	**13 drugs:** Indinavir, Niflumic acid, Saquinavir, Sulfinpyrazone, Diclofenac, Fenofibrate, Acetaminophen, Gemfibrozil, Indometacin, ALBU_HUMAN, etc.	**40 diseases:** Gilbert Disease, Hereditary Spherocytosis, Sickle Cell Anemia, Leukopenia Lymphoid Leukemia, Endometrial Stromal Tumors, Glucosephosphate Dehydrogenase Deficiency, Non Small-Cell Lung arcinoma, alpha-Thalassemia, β Thalassemia, Cystic Fibrosis, etc.
SLCO1B1 (SO1B1)	0.4576	−1.453	81	Xenobiotics	**25 drugs:** Ketoconazole, Indinavir,Glibenclamide, Saquinavir, Amprenavir, Verlukast, Ciprofibrate, Tacrolimus, Fenofibrate, Sildenafil, etc.	**1 disease:** Hepatocellular Carcinoma
CYP 2C9	0.6832	−1.219	80	Xenobiotics	**98 drugs:** Ketoconazole, Amiodarone, Metronidazole, Manidipine, Rabeprazole, Naproxen, Sulconazole, Fluvastatin, Dapsone, Tegaserod, etc.	**3 diseases:** Stomach Neoplasms, Drug Toxicity, Breast Neoplasms
CYP2D6	0.8369	−0.8532	49	Xenobiotics	**103 drugs:** Amiodarone, Betaxolol, Indinavir, Manidipine, Rabeprazole, Sulconazole, Desipramine, Clemastine, Tegaserod, Cimetidine, etc.	**1 disease:** Colorectal Neoplasms
ABCB1 (MDR1)	0.5266	−0.5369	12	Xenobiotics	**79 drugs:** Ketoconazole, Amiodarone, Manidipine, Buspirone, Clemastine, Promazine, Noscapine, Idarubicin, Laniquidar, Alimemazine, etc.	**17 diseases:** Contact Allergic Dermatitis, Stomach Neoplasms, Soft Tissue Neoplasms, Uveal Melanoma, Non-Small-Cell Lung Carcinoma, Esophageal Neoplasms, Creutzfeldt-Jakob Syndrome, Ductal Glioblastoma, Breast Carcinoma, Alzheimer Disease, etc.

**Figure 5:**
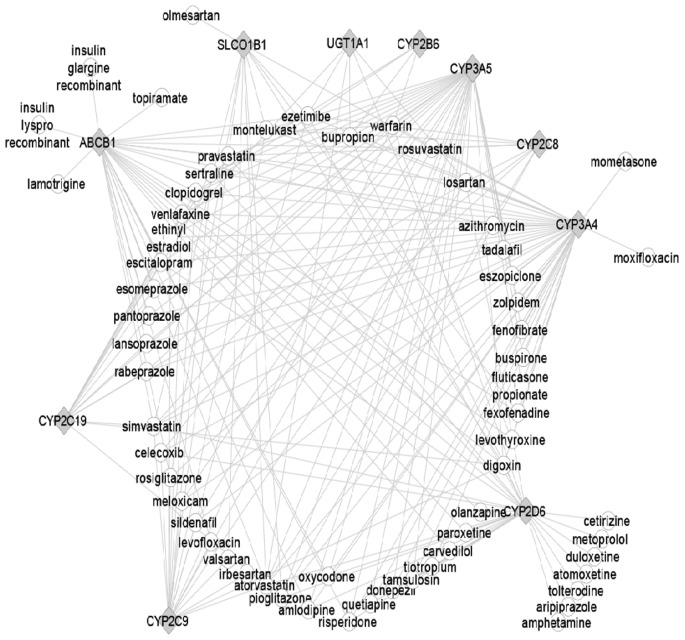
Drug-gene interaction network. The figure indicates the relationship among the ten most influential DMETs and the top 100 prescribed medications. A line between a gene and a drug suggest that the DMET is involved in the metabolism or transporting of the drug. A drug is labeled as a circle and a gene is labeled.

The top 10 most important DMET genes and the top 100 most prescribed drugs were visualized with Cytoscape (www.cytoscape.org, Figure 5). *CYP3A4, CYP3A5, CYP2C9, CYP2C19, CYP2D6 and ABCB1* are the major nodes associated with the majority of these drugs. Interindividual variability in the expression of these DMETs, an important biological significance caused by genetic variations and environmental factors of the genes, would be expected to have a large impact on individual drug metabolism, efficacy and adverse reactions.

### Nuclear receptor mediated regulation of the expression of DMETs

**Figure 6:**
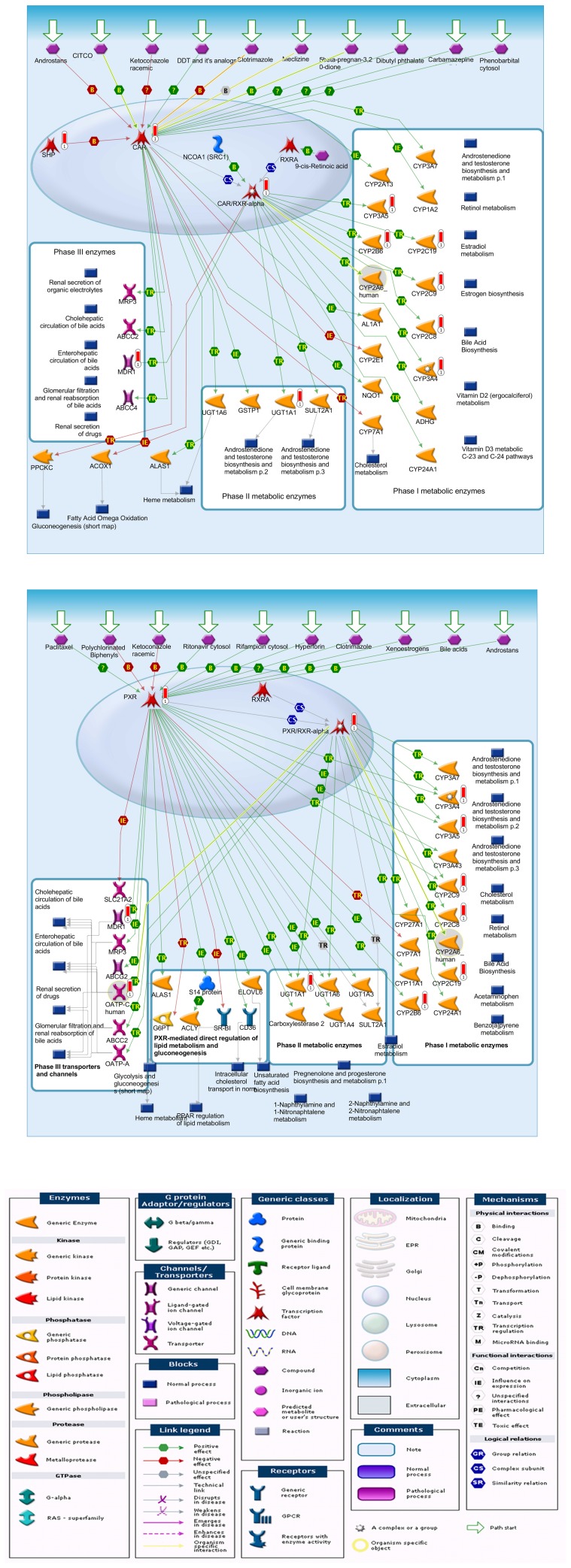
Regulation pathways for DMET expression by GeneGo analysis. The figure indicates the relationship among the ten most influential DMETs and drugs (Table 2), and the most common nuclear receptors (Table 3). The Panel A indicats the CAR/RXR mediated pathways in the regulation of DMET gene expression, and the Panel B indicates the PXR/RXR mediated pathways in the regulation of DMET gene expression. Panel C lists the legends to visualize the GeneGo pathway maps.

To investigate the mechanisms and relationships underlying the expression variability of top 10 most important DMETs (listed in the Table 2) and the expression variability of nuclear receptor genes (listed in the Table 3), we applied data analysis using GeneGo software. As shown in Figure 6, when all gene IDs of nuclear receptors (Table 3) and DMETs (Table 2) were uploaded into the Data Analysis Wizard, the enrichment analysis indicated that the constitutively expressed androstane receptor (CAR, gene symbol NR1I3) and pregnane X receptor (PXR, gene symbol NR1I2), interacting with retinoid X receptor alpha (RXRA), were pivotal mediators in the regulation of the expression of DMETs. For example, CAR/RXR mediated the expression of CYP2B6, CYP2C9, CYP2C9, CYP2C19, CYP3A4, CYP3A5, UGT1A1, etc., through binding of different xenobiotics, such as phenobarbitals, androstane and carbamazepine (Figure 6A). Similarly, PXR/RXR or PXR/PXR regulated the expression of DMETs in the Table 2, such as CYP2A6, CYP2B6, CYP2C9, CYP2C9, CYP2C19, CYP3A4, CYP3A5, CYP3A7, UGT1A1, etc., which were stimulated by different ligands such as rifampicin, polychlorinated biphenyls, and hypertorin (Figure 6B).

**Table 3:** Interindividual Variability in the Expression of Nuclear Receptor Genes among 427 Subjects.

Gene Symbol	Entrez Gene ID	Highest Expression (log10 ratio)	Lowest Expression (log10 ratio)	Expression Variability (Fold Change)
AHR	196	0.5589	−0.8767	27
ARNT	405	0.3353	−0.3176	4
ESR1	2099	0.5367	−1.187	53
HNF1A	6927	0.3912	−0.6903	12
HNF4A	3172	0.9861	−0.2267	16
IFNA	3438	NA	NA	NA
IFNR	3466	NA	NA	NA
NFE2L2	4780	0.4233	−0.5602	10
NR0B2	8431	0.9219	−0.8689	62
NR1C1	5465	0.3826	−0.3637	6
NR1H3	10062	0.4048	−0.5631	9
NR1H4	9971	0.2728	−1.052	21
NR1I1	7421	1.429	−0.8548	192
NR1I2	8856	0.4092	−1.064	30
NR1I3	9970	0.661	−2	458
NR3C1	2908	0.2928	−0.5567	7
NR5A2	2494	0.5085	−0.6075	13
PPARG	5468	2	−1.459	2877
TNF	7124	0.7351	−0.7636	32
TNFRSF11A	8792	0.6535	−0.2374	8

## Discussion

The variability in the level of expression of 374 DMET genes in 427 human livers was determined using weighted co-expression analyses (TOM, see Methods), based on a published gene expression data set [10]. The expression of 374 selected DMET genes was highly variable relative to 20 genes typically considered to have stable and less variable gene expression (i.e., housekeeping genes). Furthermore, the 374 DMETs were observed to be more variably expressed than 19,167 non-DMET genes (Figure 1). The DMETs are critical for detoxification of endogenous and exogenous compounds, and the commonly observed variability in DMET expression has been linked to risks of disease and adverse response to exogenous compounds [19], [20], [21], [22], [23]. To our knowledge, this work is the first study to comprehensively assess the variation in expression of the DMETs involved in Phase 1, 2, and 3 metabolisms.

Selected DMETs in each of the three phases of metabolism were variably expressed in the human liver samples analyzed in this study (Table 1 and Figure 2). Among the DMETs that have been well characterized previously as having highly variable gene expression and metabolic functions [24], several were observed in our study. For example, variability in the *CYP3A4* expression has been correlated with pathological processes and pharmacogenetic response to several exogenous compounds [24], [25], [26]. This gene varied by 641-fold among the samples in our study. Variation in expression of this gene would be expected to influence the metabolism of an unknown number of compounds including 245 related drugs (see www.pharmGKB.org). Other highly characterized DMETs were also observed to be variably expressed in this sample set. *CYP2B6* was associated with more than a 2,000-fold difference in expression among the samples. *CYP2B6* is responsible for metabolizing at least 57 pharmacologic agents. Among the top 20 most variable DMETs in our study, marked variation in gene expression among several members of the solute carrier transporter family (SLC) and the adenosine triphosphate-binding cassette transporter (ABC) family was also found. The extreme variability in expression for these transporters (*SLC13A1*, *SLC16A8*, *SLC16A14*, *ABCC13*, *and ABCA12*) precluded estimates of the difference in fold expression. The extreme expression phenotypes are displayed as dots in Figure 2. Members of both transporter families have been increasingly found to contribute to interindividual differences in metabolism of endogenous and exogenous compounds [27], [28], [29], [30]. These observations support further explorations of the roles of variability in the expression of various transporters in the response and clearance of endogenous and exogenous compounds.

Topological overlap matrix (TOM) analyses revealed 10 distinct modules of DMETs with highly interconnected expression patterns (Figure 3). As expected, some of the modules are comprised of DMETs that are members of extended families and/or subfamilies. For example, one module (green in Figure 4) is comprised of five uridine diphospho (UDP)-glucuronosyltransferase (*UGT*) enzymes which are created by alternative splicing or gene duplication on chromosome 2q37 (*UGT1A10*, *UGT1A4*, *UGT1A5*, *UGT1A6*, *and UGT1A9*). Co-expression of these genes would be expected. However, nine of the ten modules contained DMET genes in combinations that were less predictable. For example, one module suggests a correlation in the expression of members of the *CYP2C* subfamily (*2C9* and *2C19*) and members of the *UGT* family (*UGT1A1*, *UGT1A3*, *UGT1A7 and UGT1A8*). Members of the *CYP2* and *CYP3* families were co-expressed (blue module), and individual genes of this module (i.e., *CYP2B6*, *CYP2C8* and *CYP3A4*) contribute significantly to metabolism of some drugs. The most unexpected result was a module (turquoise) comprised of members that contribute to each phase of metabolism and the drug disposition processes (Phases 1–3). Why this set of genes appears together by TOM analyses to be co-regulated will require additional studies.

As has been observed by others [31], [32], some of the DMETs shared transcription factor binding sites with other DMETs from different enzyme families within their respective modules. For example, some DMETs from the *CYP2* and *CYP3* families (blue module) share transcription factor binding sites that are regulated by the nuclear receptor *PPARG*. *PPARG* was the most variably expressed nuclear receptor among the samples in our cohort. Moreover, additional nuclear receptors that are known to regulate expression of DMETs were also variably expressed in this data set (Table 3). Furthermore, we observed that the respective DMETs in some of the modules share transcription binding sites that have not been well characterized as contributors to drug metabolism and disposition.

The top 10 DMETs known to metabolize the most compounds are listed in Table 2. This information is striking because considerable variability in expression of the DMETs was observed in the samples analyzed, and these genes are involved in metabolism of the majority of pharmacologic interventions. *CYP3A4* has a 641-fold difference in expression among individuals in this sample set, and is involved in the metabolism of the largest number of medications. Furthermore, the DMETs that are most influential in drug metabolism have been associated with occurrence and/or pathophysiology of various diseases [31], [32], [33]. Future studies exploring the roles of DMETs in metabolizing and detoxifying endogenous and environmental compounds may help elucidate some of the unknown contributors to the pathogenesis of the associated diseases.

The drug-gene interaction network represented in Figure 5 displays the interaction of the ten most influential DMETs and the top 100 prescribed medications. This figure highlights the impact of variable DMET expression and their polymorphisms on disposition and metabolism of the most commonly used pharmacologic agents. Studies have provided evidences that DMETs are variably expressed among individuals, and genetic variants contribute to the inter-individual variability in the expression of DMETs in human liver [10], [20], [24], [34]. Previously published data identified significant associations between genotypes and the gene expression among more than 600 genes including DMETs [10]. A recent review article summarized that the expression of CYPs is regulated by multiple factors including genetic polymorphisms [34]. In addition, the expression of DMETs is also affected by other factors including sex, age and environmental exposures. To investigate of the expression viabilities of DMETs gene between sexes, our prior study analyzed the sex differences in the expression of 374 DMETs using the same dataset [10], [35]. In that study, we identified that 77 out of 374 genes showed differential expression due to sex, which is partially consistent with other reports [5], [11]. However, different conclusions for some sexually differtially expressed DMET genes were also observed, which could be due to different sample sizes in different studies [36]. Considering the cohort of 234 male and 193 female subjects in the study, our results [35] could represent more reliable conclusion of the effect of sex on DMETs expression. In term of the effect of age on the variable DMET expression, studies have been reported that age was an important variable involved in the regulation of many DMETs [11], [37]. For example, Yang *et al.* showed evidences that age impacted activities of certain CYPs [11]. Notably, the age-related correlation was relatively weak. In the future, more detailed studies are warranted to unseal the impacts of those factors on the DMET expression. This study is a comprehensive analysis of DMET expression in a large human liver cohort, but there are limitations to this analysis. The samples for this cohort were all obtained from European-Americans, and the observed expression patterns may not occur in other ancestral backgrounds or different European populations (North to south gradient in Europe - genes mirror geography within Europe [38]). Lifestyle factors (e.g., diet and physical activity) were not assessed in the study or in the analyses. Additional limitations for this cohort have been described elsewhere [11]. For example, detailed information related to donors' health status, environmental exposure history and medication records are not available for the analysis, although these donors were considered as “normal” individuals. Many dietary components and drugs could induce or inhibit the expression levels of DMET genes, thus noise could be introduced to the analyses due to the unavailability of such information.

Nuclear receptor mediated transcriptional regulation of DMETs is one of the most important mechanisms in the expression regulation of DMET genes. Generally, four ligands, including CAR (NR1I3), PXR (NR1I2), aryl hydrocarbon receptor (AhR) and peroxisome proliferator activated receptor γ (PPARγ, or NR1C3), play a key role in the induction of expression of DMETs[39], [40]. In addtion, 10 other nuclear factors, including HNF1α (hepatic nuclear factor 1), HNF4α (hepatic nuclear factor 4), NR1I1 (vitamn D receptor), NROB2 (nuclear receptor subfamily 0), NR1H4 (nuclear receptor subfamily 1), NFE2L2 (nuclear factor erythroid-derived 2), GR (glucocorticoid receptor, NR3C1), FXR (Farnesoid X-activated receptor, NR1H4), LXR (Liver X nuclear receptor α, NR1H3), LRH-1 (Liver nuclear receptor homolog-1 variant, NR5A2) are also involved in the transcriptional regulation of the DMETs [39], [40]. In this study, we searched the possible pathway that may illustrate the regulatory effects of nuclear receptors on the expression of DMETs with the GeneGo software (Figure 6). Apparently, 8 out of 10 most variably expressed DMETs (except SLCO1B1 and ABCB1) were mediated by CAR or PXR separately (panel A or B). It has been reported that both CAR and PXR activate the gene transcription of CYP2Bs, CYP2Cs, CYP3As, UGT1A1 and MDR1[41], [42]. Our observation here was consistent with the previous findings. In addition to the DMET polymorphisms, the different patient medication histories (different drugs and different dosages) could have contributed to the variable activation of different nuclear factors that in turn could affect the variability of the expression of DMETs.

In summary, a comprehensive analysis of the expression of DMETs involved in Phase 1 through Phase 3 disposition and metabolism of pharmacologic agents in a large human liver cohort was conducted. More variation in the expression of DMETs than non-DMET genes was observed, and the variability was found for genes involved in each phase of drug metabolism and disposition. Moreover, ten modules of DMETs that were coexpressed in this cohort were identified. Significant variability in expression of nuclear receptors that are known to regulate the expression of DMETs was observed. Finally, to display the clinical significance of the variability in expression of DMETs, a graphical network displaying the ten most influential DMETs and their substrates from the top 100 prescribed drugs was created. These results provide a molecular explanation to a well-known fact that drug efficacy and safety differs significantly between individuals, even among the same ancestral group (in this case, European ancestry). Predicting how gene expression may differ among individuals remains a challenge for developing personalized medicine. In addition, two important implications emerge from this analysis:

- The results provide strong evidence that individuals have different gene expression patterns adding to the variability in response expected from polymorphisms in coding regions of genes. That is, the full range of metabolism of a drug in an individual will be the combined contribution of gene expression changes often regulated by environmental factors such as naturally occurring chemicals in the diet (e.g., fatty acids, phytoestrogens, amino acids), by lifestyle such as activity levels, and by exposure to drugs and toxicants (e.g., [43]). These factors have to be measured to understand why the gene expression patterns differ so greatly between different individuals.
- In addition to gene expression changes, drug metabolism will also be influenced by differences in enzyme activity due to variation in amino acid sequences. The full array of coding SNPs in these samples were not available since genotyping reported in the article [10] was done with a 500K Affymetrix chip.

The variability in the expression of many different genes found between different individuals has significant consequences for the design of biomedical studies, including those of drug efficacy and safety. Interindividual variation in disease incidence, severity, response to medications, and outcomes has challenged traditional medical practices that rely upon data generated from prospective case-control studies. The assumption of such experimental designs was that the average response of the individuals treated in the study could be used to predict outcomes in patients in the general population. The analyses described in this report provide examples of why that assumption is not valid, because gene expression and co-expression patterns of drug metabolizing genes are highly variable and each individual may have a unique pattern of expression of these 374 DMET genes. Developing novel experimental designs that account for genetic variation and its consequences remains a challenge for the entire biomedical community.

## Methods

### Dataset

The dataset used for this analysis was from the study done by Schadt et al. [10]. A total of 427 liver samples were retrieved from three independent liver collections. The liver samples (1–2 g) were originally acquired from Caucasian individuals from three independent liver collections at Vanderbilt University, the University of Pittsburgh, and Merck Research Laboratories [10]. The Vanderbilt samples (231) included both postmortem tissue and surgical resections from organ donors. The Pittsburgh samples (171) were normal postmortem human liver, so were the Merck samples (25). Sex was well balanced for the Vanderbilt (M/F 121/110) and Pittsburgh (M/F 93/78) collections. However, the Merck collection was male biased (M/F 20/5), but its sample size was small. The average age of the donors is 52, 51, and 46 for the Vanderbilt, Merck, and Pittsburgh collections, respectively. The differences among the sample collections might have contributed, at certain degree, to the inter-individual differences observed in our analysis. Gene expression data were generated using Agilent two-color microarrays consisting of 39,302 probes corresponding to 19,541 genes, among which are 374 DMET genes (Table S1). The standard deviation (SD) and coefficient of variation (CV) for a given gene across the 427 individuals were calculated to describe interindividual variability.

### Constructing the gene coexpression network

All 374 DMET genes were chosen for constructing the weighted gene coexpression network [15]. A matrix of the pair-wise Pearson correlation coefficients was constructed before it was converted to the adjacency matrix by the function, 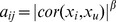, where a_ij_ denotes the connection strength between gene expressions *x_i_* and *x_j_* across 427 samples. The parameter *β* is determined in such a way that the coexpression network is approximately scale-free [17]. The model fitting index *R^2^* of the linear model that regresses *log*[*p*(*k*)] on *log*(*k*) is introduced to measure the fitting of the network to this scale-free topology, where *k* is the connectivity and *p*(*k*) is the probability density of the connectivity. A value of *β* = 7 was chosen because it achieved a fitting index greater than 0.8. The adjacency matrix is further transformed into the topological overlap matrix [16], as the topological overlap between two genes reflects not only their direct interaction but also their indirect interactions through all the other genes in the network. The average linkage hierarchical clustering was applied to group genes based on the topological overlap matrix, from which two modules were identified.

### Construction of the drug-gene interaction network

The drugs were the top prescriptions dispensed in 2006 (http://www.rxlist.com/script/main/art.asp?articlekey=79437). All commercial names were then manually transformed into their corresponding generic names before constructing the drug-gene interaction network. Genes are the top 10 most important genes in the DMET list, their importance being measured by the number of drugs that are associated with them. All the drug-gene and gene-disease relationships were retrieved from PharmGKB (http://www.pharmgkb.org), a pharmacogenomic and pharmacogenetic knowledge base and GeneGo (http://www.genego.com) software/database that consists manually-curated dada by experts, a data mining & analysis tool in systems biology. The network was visualized in Cytoscape (http://www.cytoscape.org).

### Regulation pathways for DMET expression by GeneGo analysis

Regulation pathways for DMET expression were analyzed using the GeneGo MetaCore software package. For GeneGo analysis, the gene ID number and gene expression data were uploaded into Data Analysis Wizard (General parser), Homo Sapiens was selected as the specie. After background processing, the file was analyzed with enrichment analysis workflow tool, resulting in the list of most significantly enriched pathways.

## Supporting Information

Table S1 **The inter-individual variability of 374 DMETs among 427 human liver samples.**
(XLS)
